# Interventions for preventing or treating malnutrition in problem drinkers who are homeless or vulnerably housed: protocol for a systematic review

**DOI:** 10.1186/s13643-015-0114-3

**Published:** 2015-09-29

**Authors:** Helen Thorley, Katie Porter, Clare Fleming, Tim Jones, Joanna Kesten, Elsa Marques, Alison Richards, Jelena Savović

**Affiliations:** The National Institute for Health Research Collaboration for Leadership in Applied Health Research and Care West (NIHR CLAHRC West) at University Hospitals Bristol NHS Foundation Trust, 9th Floor Whitefriars, Lewins Mead, Bristol, BS1 2NT UK; School of Social and Community Medicine, University of Bristol, Bristol, UK; Bristol City Council, St Anne’s House, St Anne’s Road, Bristol, BS4 4AB UK; Compass Health, The Compass Centre, 1 Jamaica Street, Bristol, BS2 8JP UK

**Keywords:** Homeless, Alcoholism, Alcohol, Dependence, Malnutrition, Nutrition, Micronutrients, Deficiency, Thiamine, Systematic review, Intervention, Supplements

## Abstract

**Background:**

Problem alcohol drinking in homeless and vulnerably housed people can lead to malnutrition, which is associated with complications such as alcohol-related brain damage. Homeless alcohol drinkers are likely to have worse health outcomes and different nutritional needs compared with housed alcohol-drinking persons. It is not clear whether interventions to improve nutritional status in this population have been effective. The purpose of this review is to assess the effectiveness and cost-effectiveness of interventions for preventing or correcting micronutrient deficiencies and other forms of malnutrition and related comorbidities in this population.

**Methods/design:**

A systematic search for studies of a nutrition-based intervention applied in the homeless or vulnerably housed population with problem drinking will be conducted. The following electronic databases will be systematically searched for relevant studies: MEDLINE, EMBASE, Web of Science, PsycINFO, CAB abstracts, CINAHL, Cochrane Public Health Group Register and Cochrane Drugs and Alcohol Group Register. Screening of identified abstracts for relevance and assessment of papers for inclusion will be done in duplicate. One reviewer will extract data from the studies and assess quality, and this will be checked by another reviewer. Discrepancies will be resolved by consensus. The primary outcomes are (mal)nutrition status or micronutrient deficiencies or change in (mal)nutrition status or micronutrient deficiencies, measures of liver damage and cognitive function. Secondary outcomes include comorbidities, quality of life and functional scales, resources used to deliver treatment, uptake/acceptability of the intervention and engagement with treatment services. Results will be analysed descriptively, and, if appropriate, meta-analyses will be performed.

**Discussion:**

The results of this review should help to inform the development of effective interventions that can be implemented in the community to improve the health of homeless people who are problem drinkers.

**Systematic review registration:**

PROSPERO CRD42015024247

**Electronic supplementary material:**

The online version of this article (doi:10.1186/s13643-015-0114-3) contains supplementary material, which is available to authorized users.

## Background

Problem drinking is an important problem among the homeless population in developed countries. Estimates range from 8 to 58 %, with the prevalence increasing in more recent years [1, 2]. In the UK, 35 % of the homeless drink twice per week or more with around 63 % of the homeless drinking more than the recommended limit every time [3, 4]. Official estimates for the number of rough sleepers in England vary between about 2500 and 8000, with this figure on the rise [5, 6]. However, some form of homelessness applies to a much wider population. This is difficult to measure, with the ‘hidden homeless’ staying with friends or family, in hostels or bed and breakfasts or in other vulnerable housing situations [7].

Problem drinkers who are homeless tend to obtain a large proportion of their energy intake from alcohol, and as a result, they are rarely underweight [8, 9]. However, this alcohol-rich diet is likely to lack a variety of important nutrients including micronutrients such as vitamins and minerals as well as macronutrients such as protein [10, 11]. Alcohol causes damage to the gastrointestinal tract, reducing effective absorption of consumed nutrients such as sodium, carbohydrates, water, proteins and fats [12]. This damage often results in gastrointestinal motility issues and digestive disorders. These in turn cause problems, such as loss of appetite, nausea, vomiting, diarrhoea, abdominal pain, maldigestion and malabsorption of nutrients, all of which may contribute to malnutrition [12]. Thiamine deficiency in particular can impair cognitive function and make behaviour change, such as abstinence from drinking, much more difficult to achieve [13]. If untreated, malnutrition in heavy drinkers can lead to serious complications such as brain damage (Wernicke’s encephalopathy and Korsakoff’s psychosis), liver disease, impaired immunity and infections [14–16].

People experiencing homelessness are at risk of being malnourished due to a low income and lack of ways to store and cook nutritionally beneficial foods, leading to a reliance on cheap, ready-prepared foods [17]. They may also lack knowledge of how to achieve a healthy diet in their situation. Provided food sources (e.g. soup kitchens) make an important contribution to energy intake and nutritional health [17], but may not be optimised to improve users’ diets. Limited resources and staff training preclude the provision of fresh, high-quality food sources [10, 11, 18]. Often, the goal of charitable food providers is to provide filling meals to stave off hunger rather than improving nutritional health [19]. Persons who are both homeless and problem drinking simultaneously are at a particularly high risk of malnutrition as difficulties in accessing nutritional food are compounded by excessive alcohol consumption. Severely dependent drinkers with a low income might choose to spend their available money on alcohol instead of food [20]. Additionally, homeless people do not receive the benefit of household members (e.g. family) who can influence the eating behaviours of housed heavy drinkers [21–23]. Given the combined effects of homelessness and heavy alcohol drinking upon the risk of malnutrition, homeless problematic drinkers are likely to have different nutritional needs from non-homeless individuals.

Improving the nutritional status of homeless heavy drinkers might be achieved by adapting the dietary intake of participants to be more nutritionally beneficial, either through informing or encouraging participants to change their dietary choices or by improving the nutritional quality of the food available to them. Interventions to achieve this may include distribution of a specific fortified food product or supplement; interventions delivered through soup kitchens (e.g. menu changes, fortification of food, etc.); advice to care providers or food providers on how to tackle deficiencies; or peer support interventions related to nutrition. Specific interventions proposed in the literature include a chocolate spread fortified with micronutrients specifically developed for the homeless population in Paris [24], nutrition classes delivered to homeless mothers and cafeteria staff [25], leaflets for the homeless aimed at improving nutritional literacy [8] and guidelines for a daycare centre providing food to the homeless [26].

### Why it is important to do this review

The aim of this review is to bring together existing evidence of the effectiveness of nutritional interventions in homeless drinkers, in order to understand how the nutritional status of this population could be improved. This review will inform the development of effective interventions that can be implemented in the community to improve health of the target population.

Systematic reviews ascertaining the effectiveness of interventions to improve health in the homeless have been undertaken. The focus of these reviews was on interventions that tackle either the housing problem or substance abuse [27–29], rather than nutrition. The current review will identify whether there are effective and affordable ways to improve nutrition directly or indirectly by increasing the likelihood of complex behaviour change such as alcohol detoxification. Thiamine deficiency in particular can impair cognitive function and make behaviour change much more difficult to achieve [13]. Similarly, reviews addressing nutrition in problem alcohol drinkers exist [30, 31], but problem drinkers who are homeless or otherwise in vulnerable housing represent a special case. Intervention to address problematic drinking will not necessarily be applicable to problematic drinkers who are also homeless. Studies regarding the non-alcohol-drinking homeless, while still relevant, will also not necessarily apply to the alcohol-drinking homeless. This review aims to bring these two elements together and look at the interventions aimed at improving nutritional status of problem drinkers that can be successfully delivered to the homeless population.

### Objective

The objective of this review is to assess the effectiveness and cost-effectiveness of interventions for preventing or correcting micronutrient deficiencies and other forms of malnutrition and related comorbidities in problem drinkers who are homeless or vulnerably housed.

## Methods/design

### Criteria for considering studies for this review

#### Types of studies

Homeless populations are not well-suited to large-scale randomised controlled trials (RCTs). Nutritional interventions are expected to be delivered though points of contact with the homeless—charity organisations, soup kitchens, etc. Therefore, we anticipate that there may be limited evidence from RCTs which assess the benefits, risks and outcomes of nutritional interventions.

We will include the following study designs: RCTs, including cluster RCTs; non-randomised controlled studies (e.g. studies with multiple clusters/communities where allocation to interventions was not randomised); controlled before and after studies; and interrupted time-series studies [32]. Initially, we plan to exclude uncontrolled before and after studies. We will however review this decision after assessing all literature for eligibility. If this strategy returns a small number of included studies, we may revise the inclusion criteria to include uncontrolled studies. Studies with either individual or aggregate data collection will be included. Opinion pieces, editorial articles, case studies and qualitative studies will be excluded.

#### Types of participants

The participants of this review are any problem drinkers who are experiencing homelessness or who are in a vulnerable housing situation. We will define homelessness based on the legal definition used in the UK, which includes the following: sleeping rough (outside); residing in temporary accommodation such as hostels, bed and breakfasts or night shelters; staying on a temporary basis with family or friends (‘sofa surfers’); currently housed people who are at risk of being evicted; and currently housed people who cannot stay because they cannot afford to stay, the home is in a very poor condition or they are subject to violence, abuse or threats in the home [33].

Inclusion of studies with a mix of homeless and non-homeless populations will be decided on a case-by-case basis. Studies will be included if the majority of participants are homeless according to the above definition. We will exclude studies for which the entire communities are homeless (e.g. occupiers of slums or shanty towns, refugees or similar populations). We will also exclude those who are homeless for reasons of mass displacement as a consequence of catastrophic events such as war, famine or natural disaster, as it is expected that the characteristics and logistics of delivering interventions will be markedly different in these groups.

The definition of problem drinking will not be confined to a diagnosed alcohol addiction but includes any heavy or problem drinking, as defined in included studies. All definitions of heavy or problem drinking will be included, and the definition will be recorded.

Studies involving participants with other substance abuse will be included if this is in addition to alcohol abuse, either at the individual level or within the group. We expect that there may be complementary effects between different types of substance abuse, and we do not want to exclude relevant studies that do not report data for alcohol abusers separately.

We will include studies in homeless populations that do not separately report data for problem drinking populations as the evidence suggests that there is a relatively high proportion of problem drinkers among the homeless population [3, 34]. In addition, studies which focus on homeless populations, regardless of the proportion of alcohol drinkers, will be useful to inform intervention delivery to this population, which is expected to be challenging. We will however exclude studies in the homeless population if they specifically exclude heavy or problem drinkers.

#### Types of interventions

Studies will be included if they contain an intervention that aims to improve the nutritional status or macro- or micronutrient deficiencies in the population of interest. The intervention may be a process or a product and could involve, but is not limited to, the following: the distribution of food or supplements (including specific fortified food products, food supplements, single or multiple micronutrient supplements or food in general); improved access to food (including specific fortified food products, food supplements or food in general); information or training provision (i.e. advice delivered to participants to improve nutritional education); interventions delivered through food providers such as soup kitchens or food banks (e.g. menu changes, fortification of food, etc.); advice to care providers or food providers on how to address deficiencies; peer support interventions related to nutrition; or medical interventions such as oral or parenteral micronutrient supplementation (e.g. parenteral thiamine).

Interventions without any nutritional, food-related component or micro- or macronutrient supplementation (e.g. interventions solely aimed at improving the housing status of individuals, peer support or advice focused on stopping drinking, interventions focused solely on reducing alcohol intake) will be excluded.

#### Types of outcome measures

We will not exclude articles based on the outcome measures reported. All studies with eligible participants and interventions will be included, regardless of outcome measures reported. See the ‘Data extraction’ section for primary outcomes and a full list of outcomes that will be extracted.

### Identification of studies

#### Electronic searches

The following databases will be searched using combined search terms and subject headings relating to homelessness, problem drinking and nutrition: MEDLINE, EMBASE, Web of Science, PsycINFO, CAB abstracts, CINAHL, Cochrane Public Health Group Register and Cochrane Drugs and Alcohol Group Register. The full search strategy for MEDLINE is available and illustrates the planned search strategy for the electronic database searches (Additional file 1). The search is conducted from inception of the databases. We will not apply any language restrictions. If articles identified are written in a language other than English, we will commission their translation into English.

#### Searching other sources

Key organisations in the area of health and nutrition will be contacted and, if applicable, their websites searched. These key organisations will include the following: Alcohol Concern [35], The Society for the Study of Addiction [36], Alcohol Research UK [37], The Faculty for Homeless and Health Inclusion [38], The National Institute of Alcohol Abuse and Alcoholism [39], Trussell Trust [40], SMART recovery programme [41], Department of Health for England and other countries, The Medical Council on Alcoholism [42], Shelter [43], The Salvation Army [44], Homeless link [45], Crisis [46], and Alcoholics Anonymous [47].

A search engine approach will be used, by manually searching the first three pages of results for key search terms. Additionally, the reference lists of included studies and relevant systematic reviews will be checked for potentially relevant studies.

For assistance in identifying ongoing or unpublished studies, we will contact key experts as well as authors of included studies and ask them to identify unpublished or ongoing research in the field.

### Data collection and analysis

#### Selection of studies

Upon the removal of duplicates, the citation records from the electronic database searches will be imported into a custom-designed Microsoft Access 2013 database. The titles and abstracts will be independently screened in duplicate to assess if the article is potentially relevant. Wherever a title or abstract cannot be rejected with certainty, it will be classed as potentially relevant, and all potentially relevant articles will be taken through to the next stage of screening.

Full-text manuscripts for all potentially eligible articles will be obtained and read in full. All full-text manuscripts will be assessed in duplicate for eligibility against the inclusion and exclusion criteria. Disagreements related to the inclusion of articles will be resolved through discussion and consultation with a third reviewer, where necessary. Studies excluded at this stage will be documented along with reasons for exclusion. A draft study selection form is provided (Additional file 2).

#### Data extraction

Data will be extracted for all studies meeting the inclusion criteria using a pre-specified and piloted data extraction form. The following information will be extracted: bibliographic information; study details including aim of the study; study characteristics; patient characteristics; sample size; measures of quality; which outcomes are reported; and outcome data including treatment effect estimates, *p* values and confidence intervals. Data will be extracted by one reviewer and checked for accuracy and completeness by a second reviewer. Discrepancies will be resolved through discussion (with a third reviewer where necessary). Where there are multiple reports of the same study, these will be treated as a single study and all available sources will be used for data extraction. Where data is unclear, we will attempt to contact the authors for clarification.

The following study characteristics will be extracted: country, year, settings (self-catered or non-self-catered hostel, soup kitchen, food bank, etc.), and where the food is provided from. Patient characteristics will include the following: gender; age; co-addiction; stage of disease/treatment (active drinking, in treatment, dry, etc.); baseline nutritional profiles; duration and categories of homelessness; and ethnic group. We will also record if any of these or other characteristics have been found to be effect modifiers in the original studies.

The primary outcomes for this review are malnutrition status, improvements in macro- or micronutrient deficiencies (individual or combined) and related comorbidities. Comorbidity, although not directly attributable to malnutrition, is an important marker of nutrition status. Thus, data will be collected for a defined list of comorbidities and other outcomes. Specifically, we will collect data for the following outcomes if available:

- (Mal)nutrition status or micronutrient deficiencies or change in (mal)nutrition status or micronutrient deficiencies (likely to be in the form of a dietary assessment or blood tests)
- Measures of liver damage (alanine transaminase, aspartate transaminase) or bone marrow damage (mean corpuscular volume)
- Functional scales of cognitive function (including memory loss)

Secondary outcomes include the following: mortality, suicide, presence of acute or chronic gastritis incidence, presence of acute or chronic pancreatitis, quality of life or well-being measures, and abstinence. We will also collect the following process outcomes: engagement with treatment services or recovery groups (including planned detoxification); planned and unplanned hospital admissions; number of people in sustained housing; acceptability of the intervention (e.g. palatability of the intervention food product); and resources or resource use required to deliver these interventions (e.g. hours of work, equipment/location use, product manufacturing costs).

Because we expect that studies will report a variety of diverse outcomes, we will also record all reported outcomes (in addition to those listed above) that each study has measured and reported, without extracting result data. Upon completion of data extraction, the team will review the nature and frequency of these additional outcomes. If particular outcomes are found to be important or consistently reported across the literature, we will consider extracting the results for these outcomes. This will be decided by consensus in the research team.

#### Assessment of risk of bias

The Cochrane risk of bias tool will be used for any RCTs which are included in the analysis [48, 49]. The risk of bias criteria listed by the Cochrane Effective Practice and Organisation of Care (EPOC) Group will be used for other types of studies [50]. This specifies standard criteria for non-randomised controlled trials, controlled before and after studies and interrupted time-series studies. Risk of bias will be assessed at the study level or outcome level, as appropriate.

#### Measures of treatment effect

Our narrative summary will report health-related outcomes as provided in the included studies. For dichotomous data, we will present proportions of participants who experience the outcome event. Where studies report two-group comparisons, we will present the results as a summary odds ratio along with 95 % confidence intervals. We will report results for continuous outcomes as the mean for each group as well as the mean difference between groups with 95 % confidence intervals. For studies that report change from baseline, we will report the mean change for each group as well as the mean difference in change between groups with 95 % confidence intervals. Where necessary, standardised mean difference may be calculated to allow comparisons across studies with different continuous measures.

#### Unit of analysis issues

The expected nature of interventions means that any trials are likely to be cluster randomised trials. If the authors have not taken account of this, an attempt will be made to investigate the risk of bias for these.

#### Dealing with missing data

If data is missing that is required for data synthesis from any included study, we will attempt to contact the authors to obtain the missing data. This includes missing data on the methods used, the outcomes or precision measures.

#### Data synthesis

The selection process and reasons for exclusion will be presented in a PRISMA flow diagram. The findings will be presented largely via summary tables and a narrative summary. Depending on the quantity and diversity of studies retrieved, we will consider different levels of grouping: intervention type, study design and outcome. We will describe findings narratively within groupings and comment on sources and implications of methodological heterogeneity across studies.

Outcome data will be extracted and presented in tables describing and summarising the results of each study. The tables will include, where appropriate and available, the following: intervention type; study type; description of the interventions (along with a list of nutrients included, where appropriate); year published; geographical location; number of participants; measures of effectiveness reported with numerical findings and precision measures; units of resource use required to deliver treatment; measures of cost-effectiveness reported with numerical findings and precision measures; acceptability of interventions; micronutrient deficiencies; and (mal)nutrition profiles.

A quantitative meta-analysis will be conducted if enough studies are found that are sufficiently similar in intervention and outcomes, for treatment effects to be synthesised. The meta-analysis will follow Cochrane methodology guidelines [49]. The software that will be used for meta-analysis is Stata V13.1 for Windows [51].

#### Investigation of heterogeneity

If meta-analyses are undertaken, we will inspect the variability in the study effect estimates and the overlap of confidence intervals. We will perform the formal *χ*^2^ test for heterogeneity and calculate the *I*^2^ statistic. If plausible, we will consider investigating heterogeneity in subgroup analyses by the type of intervention, settings or type of included population (e.g. if studies vary by the type of homeless population or proportion of heavy drinkers).

#### Sensitivity analysis

We will consider conducting sensitivity analyses if issues arise from the decisions made during the review process. If it is unclear whether studies should have been included, we may carry out a sensitivity analysis to examine the effects of removing these studies from the meta-analysis. We will perform a sensitivity analysis that only includes randomised controlled trials as the highest grade of evidence.

### Amendments to the protocol

Any changes to this protocol will be recorded and can be made available upon request.

## Discussion

This review aims to summarise the available evidence base of the effectiveness and cost-effectiveness of interventions to address malnutrition in the homeless or vulnerably housed and problem drinking population.

It is likely that the quantity of evidence for nutrition-based interventions in the target population will be limited. In particular, we do not expect studies of randomised controlled trials to be numerous. This is partly due to inherent problems with conducting trials in the homeless population, which is by nature transient and thereby creating difficulties with collecting follow-up data. As a result, we are not limiting our search to RCTs but including a range of study designs. A limitation of this review may be that results of the studies cannot be combined in a meta-analysis.

Given the expected scarcity of evidence, the absence of language restrictions and extensive grey literature search are particularly advantageous for this review. Additionally, a scoping search and pilot screening have been undertaken to refine the selection procedure, and these indicate that the number of hits to be screened should be manageable.

Another strength of this review lies in its broad scope, without strict constraints imposed on the types of outcomes or interventions considered. This will allow an exploration of the range and diversity of interventions that have been evaluated. The results of this review should help to inform the implementation of similar interventions in the community to improve the health of the problem drinking homeless.

## Additional files

Additional file 1:
**Search strategy—Medline (Ovid).** A detailed search strategy for Medline. (245 KB) Additional file 2:
**Draft study selection form.** A draft study selection form used for inclusion/exclusion of studies. (267 KB)

## Abbreviations

EPOC: Effective Practice and Organisation of Care
RCT: randomised controlled trial
