# Supplementary material for: Interventions for preventing or treating malnutrition in problem drinkers who are homeless or vulnerably housed: protocol for a systematic review
Source: Syst Rev. 2015 Sep 29;4:131. doi: 10.1186/s13643-015-0114-3 (PMC4589081; doi:10.1186/s13643-015-0114-3)
Supplement: Additional file 1: — Search strategy—Medline (Ovid). A detailed search strategy for Medline. (245 KB) [file 13643_2015_114_MOESM1_ESM.pdf]

Additional file 1: Search strategy- Medline (Ovid)

1 exp Homeless Persons/ (6848)
2 homeless$.ti,ab. (6708)
3 (rough adj2 (sleep$ or living)).ti,ab. (33)
4 ((street or streets) adj4 (drinker$ or sleeper$ or living)).ti,ab. (196)
5 ((vulnerabl$ or temporar$ or marginally) adj3 (accommodated or accommodation or housed or housing)).ti,ab. (251)
6 (hostel$ or flophous$ or flop-hous$ or dosshous$ or doss-hous$ or skid-row or "bed and breakfast$" or sofa surf$ or nightshelter$).ti,ab. (787)
7 (evict$ adj4 (home$ or hous$ or accommodat$)).ti,ab. (25)
8 social marginalization/ (130)
9 (social$ adj3 (marginali$ or exclude$ or exclusion)).ti,ab. (1427)
10 or/1-9 (11003)
11 *Substance-related disorders/co, dh, pa, pp, pc, rh, th (23198)
12 Alcoholics/ (159)
13 Alcohol-Related Disorders/co, pa, pp, pc, rh, th (1550)
14 Alcohol-Induced Disorders/co, dh, pa, pp, pc, rh, th (105)
15 drinking behavior/ and alcohol$.mp. (684)
16 exp Alcohol Drinking/co, pa, pp, pc, th (6083)
17 (wernicke encephalopathy/ or korsakoff syndrome/) and alcohol$.mp. (810)
18 Alcoholic Intoxication/co, dh, pa, pp, pc, rh, th (4179)
19 Alcoholism/co, dh, pa, pp, pc, rh, th (38520)
20 *Alcohol-Related Disorders/ or *Alcohol-Induced Disorders/ or *Alcoholic Intoxication/ or *Alcoholism/ (60148)
21 (alcohol adj2 (abus$ or misus$ or problem or problems or problematic or addict$ or dependen$)).ti,ab. (29784)
22 (alcoholic adj2 (patient$ or intoxication)).ti,ab. (7428)
23 ((heavy or binge or problem or problems or problematic or excessiv$ or dependen$) adj2 (drinker$ or drinking)).ti,ab. (12369)
24 (alcoholics or drunkard$).ti,ab. (13520)
25 alcoholism.ti. (11688)
26 ((wernicke$ or korsakoff) adj12 alcohol$).ti,ab. (515)
27 or/11-26 (118371)
28 10 or 27 (128233)
29 exp Deficiency Diseases/dh, pc, rh, th (10267)
30 Malnutrition/dh, pc, rh, th (2610)
31 starvation/dh, pc, rh, th (301)
32 *diet/ or energy intake/ (79646)
33 Food Assistance/ or food habits/ or food storage/ or cooking/ (31777)
34 food/ or bread/ or cereals/ or exp dairy products/ or dietary carbohydrates/ or dietary proteins/ or eggs/ or exp food, preserved/ or fruit/ or exp meat/ or nuts/ or raw foods/ or seeds/ or vegetables/ or vegetable proteins/ or exp dietary supplements/ or foods, specialized/ or exp food, fortified/ or cacao/ or exp meals/ (323340)
35 exp Micronutrients/ad, ut, tu, th (109906)
36 hunger/ or appetite/ or nutritive value/ (18769)
37 feeding behavior/ or food preferences/ (49834)
38 Nutritional Requirements/ (17575)
39 food services/ or menu planning/ or food supply/ or food quality/ (14757)
40 Nutrition Disorders/dh, pc, rh, th (4293)
41 nutrition assessment/ or exp nutrition surveys/ (26077)
42 ((malnourish$ or malnutrition) adj3 (prevent$ or alleviat$ or treat$)).ti,ab. (1399)

43 ((nutrition$ or malnutrition) adj3 (assess$ or profil$)).ti,ab. (7675)
44 ((nutrition$ or nutritive) adj2 value).ti,ab. (4132)
45 ((nutrition$ or nutrient$) adj3 (intake or adequate or adequacy or inadequate or inadequac$ or deficient or deficienc$ or quality or composition or improvement$ or supplement$ or assessment$ or risk or status or state or healthy or evaluation$)).ti,ab. (58331)
46 ((menu$ or meal$ or diet$) adj3 (information or education or advice)).ti,ab. (5176)
47 ((menu$ or meal$) adj3 (charit$ or low-cost or cheap$ or balanced or nutritious or quality or provide$ or provision or composition or planning or healthy or improvement$ or preference$)).ti,ab. (2617)
48 (diet$ adj3 (healthy or balanced or nutritious or improvement$ or inadequate or adequate or adequacy or deficient or deficienc$ or inadequac$ or supplement$ or better or preference$)).ti,ab. (48165)
49 ((food or foods or foodstuffs) adj3 (cooked or storage or tinned or canned or functional or parcel$ or pack or packs or assistance or choice$ or provide$ or provision or adequate or adequacy or inadequate or inadequac$ or aid or program$ or charit$ or supplement$ or high energy or fortified or healthy or cost or quality)).ti,ab. (23031)
50 (eating adj2 (well or healthily or cheaply or advice or better or regular$ or healthy)).ti,ab. (3614)
51 (multimicronutrient$ or multi-micronutrient$ or micronutrient$ or micro-nutrient$ or multinutrient$ or multi-nutrient$).ti,ab. (8760)
52 ((vitamin$ or multivitamin$ or mineral$ or benfotiamine) adj3 (pill$ or tablet$ or supplement$)).ti,ab. (17220)
53 ((chocolate or cereal or energy) adj2 (snack$ or "bar" or "bars")).ti,ab. (368)
54 (snack$ adj3 (system$ or healthy or fortif$)).ti,ab. (163)
55 (soup kitchen$ or free meal$ or cooking or eatwell or eat-well or meal service$ or regular meal$).ti,ab. (8556)
56 (thiamin$ adj2 (supplement$ or parenteral or injection$ or pill$ or tablet$ or repletion)).ti,ab. (409)
57 ((caloric or calorie$ or carbohydrate$ or protein or energy) adj2 intake).ti,ab. (29335)
58 (dietary adj2 (protein$ or carbohydrate$ or supplement$)).ti,ab. (23204)
59 ((consum$ or eat$) adj2 (fruit$ or vegetable$ or cereal$ or chocolate$ or egg$ or bread or yoghurt or nut or nuts or dairy product$)).ti,ab. (8899)
60 or/29-59 (652567)
61 28 and 60 (3889)
62 letter/ (912491)
63 editorial/ (368819)
64 news/ (165754)
65 exp historical article/ (340653)
66 Anecdotes as topic/ (4692)
67 comment/ (614119)
68 case report/ (1760670)
69 (letter or comment$).ti. (98002)
70 animals/ not humans/ (3986044)
71 exp Animals, Laboratory/ (755008)
72 exp Animal Experimentation/ (6770)
73 exp Models, Animal/ (445300)
74 exp rodentia/ (2773028)
75 (rat or rats or rodent$ or cat or cats or pig or pigs or dog or dogs or sheep or cow or cows or ovine or porcine or canine or feline or primate$ or monkey$ or mouse or mice).ti. (1549185)
76 or/62-75 (8138094)
77 exp child/ or exp newborn/ or (p?ediatric or f?etal or f?etus or pre-natal or prenatal or child$ or infant$ or neonat$ or newborn$ or baby or babies).ti. (2240520)
78 exp adult/ or adult$.ti. (5934108)
79 77 not 78 (1522181)
80 76 or 79 (9231431)
81 61 not 80 (2518)
