# Supplementary material for: Interventions for preventing or treating malnutrition in problem drinkers who are homeless or vulnerably housed: protocol for a systematic review
Source: Syst Rev. 2015 Sep 29;4:131. doi: 10.1186/s13643-015-0114-3 (PMC4589081; doi:10.1186/s13643-015-0114-3)
Supplement: Additional file 2: — Draft study selection form. A draft study selection form used for inclusion/exclusion of studies. (267 KB) [file 13643_2015_114_MOESM2_ESM.pdf]

Additional file 2: Draft Study Selection Form

Citation Details

Assessor name………………………………………. Date …………………………….

Report ID ………………….. First author ……………………………………………………..

Journal ………………………………………………………… Publication year ………………..

Study/Trial name (e.g "UK 2001-2003") ……….…………………………………………….

Study Selection Criteria

1. POPULATION: Does this paper contain a majority of homeless or vulnerably housed participants (or consider them as a subgroup). [Note: exclude those who are homeless for reasons of mass displacement or where entire populations are homeless (see definition).] yes / no

    1a. Does this paper suggest that it contains at least half alcohol dependent participants (or mixed alcohol and drug dependents)? [Note: include studies which report results separately for them (see definition).] yes / no / unclear [Don't exclude papers based on this Q]

2. INTERVENTION: Does this paper contain any nutritional-based intervention, targeted at improving nutrition, or correcting or preventing malnutrition, in any arm? [Note: Include micro- and macro-supplementation in oral or parenteral formats.] yes / no

3. STUDY DESIGN: Is the study design one of the eligible designs (RCTs; Cluster RCTs; non-randomised, controlled study; controlled before and after study; interrupted time-series)? yes / no

    3a. What is the study design?
    RCT; Cluster RCT; non-randomised, controlled study; controlled before and after study; interrupted time-series, uncontrolled before and after study, cohort study, systematic review, anecdotal evidence, case study or other *(specify)*………………………………

Status of Study
Excluded / Included / Pending

If excluded, main reason for exclusion (put 1, 2 or 3, for the 1st question answered No to): ………………
